# (Benzene­carbothio­amide-κ*S*)­penta­carbonyl­tungsten(0)

**DOI:** 10.1107/S1600536811008579

**Published:** 2011-03-12

**Authors:** Salah Merniz, Mahiedine Mokhtari, Sofiane Bouacida, Lahcène Ouahab, Abdelhamid Mousser

**Affiliations:** a Département de Chimie, Faculté des Sciences Exactes, Université Mentouri Constantine, Route de Ain El Bey, Constantine, Algeria; b Département Sciences de la Matière, Facult des Sciences Exactes et Sciences de la Nature et de la Vie, Universit’e Larbi Ben M’hidi, Oum El Bouaghi 04000, Algeria; c Unité de Recherche de Chimie de l’Environnement et Moléculaire Structurale, CHEMS, Université Mentouri-Constantine, 25000 Algeria; d Equipe Organométallique et Matériaux Moléculaires, UMR6226 CNRS-Université de Rennes 1, Avenue du Général Leclerc, 35042, Rennes, France

## Abstract

The asymmetric unit of the title complex, [W(C_7_H_7_NS)(CO)_5_], comprises two independent mol­ecules. In each, the W atom is coordinated by five CO groups and the S atom of the benzencarbothioamide ligand in a distorted octa­hedral geometry. The crystal packing can be described as undulating layers of W(CO)_5_ and benzene­carbothio­amide parallel to (001). In the crystal, components are linked *via* inter­molecular N—H⋯O and C—H⋯O hydrogen bonds to form a dimeric chains along the [010] direction. Intra­molecular N—H⋯C inter­actions are also observed.

## Related literature

For applications of thio­amides, see: Gok & Cetinkaya (2004 ▶). For the preparation of metal complexes of thio­nes, see: Raper (1994 ▶, 1996 ▶, 1997 ▶). For related structures, see: Saito *et al.* (2007 ▶); Pasynsky *et al.* (2007 ▶); Darensbourg *et al.* (1999 ▶). For the coordination characteristics of thio­amides, see: Raper *et al.* (1983 ▶)
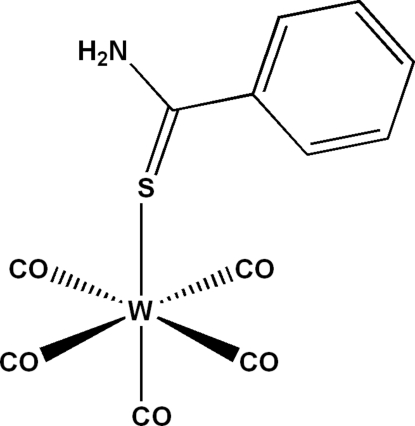


## Experimental

### 

#### Crystal data

[W(C_7_H_7_NS)(CO)_5_]
*M* *_r_* = 461.10
Monoclinic, 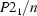

*a* = 7.311 (1) Å
*b* = 19.567 (2) Å
*c* = 20.342 (1) Å
β = 91.85 (1)°
*V* = 2908.5 (5) Å^3^
*Z* = 8
Mo *K*α radiation
μ = 8.10 mm^−1^
*T* = 295 K
0.05 × 0.05 × 0.04 mm

#### Data collection

Nonius KappaCCD diffractometer
11978 measured reflections
6616 independent reflections
5239 reflections with *I* > 2σ(*I*)
*R*_int_ = 0.039

#### Refinement

*R*[*F* ^2^ > 2σ(*F* ^2^)] = 0.043
*wR*(*F* ^2^) = 0.122
*S* = 1.09
6616 reflections
362 parameters
H-atom parameters constrained
Δρ_max_ = 2.05 e Å^−3^
Δρ_min_ = −1.81 e Å^−3^

### 

Data collection: *COLLECT* (Nonius, 1998 ▶); cell refinement: *SCALEPACK* (Otwinowski & Minor, 1997 ▶); data reduction: *DENZO* (Otwinowski & Minor, 1997 ▶) and *SCALEPACK*; program(s) used to solve structure: *SIR2002* (Burla *et al.*, 2003 ▶); program(s) used to refine structure: *SHELXL97* (Sheldrick, 2008) ▶; molecular graphics: *ORTEP-3* (Farrugia, 1997 ▶) and *DIAMOND* (Brandenburg & Putz, 2001 ▶); software used to prepare material for publication: *WinGX* (Farrugia, 1999 ▶).

## Supplementary Material

Crystal structure: contains datablocks global, I. DOI: 10.1107/S1600536811008579/zk2003sup1.cif

Structure factors: contains datablocks I. DOI: 10.1107/S1600536811008579/zk2003Isup2.hkl

Additional supplementary materials:  crystallographic information; 3D view; checkCIF report

## Figures and Tables

**Table 1:** Hydrogen-bond geometry (Å, °)

*D*—H⋯*A*	*D*—H	H⋯*A*	*D*⋯*A*	*D*—H⋯*A*
N1*A*—H2*A*⋯O3*B*^i^	0.86	2.52	3.174 (8)	133
C9*B*—H9*B*⋯O4*B*^ii^	0.93	2.53	3.285 (10)	139
N1*B*—H2*B*⋯C3*B*	0.86	2.59	3.263 (9)	136
N1*A*—H2*A*⋯C4*A*	0.86	2.69	3.412 (10)	142

Symmetry codes: (i) 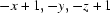; (ii) 
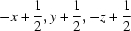
.

**Fig. 1.**
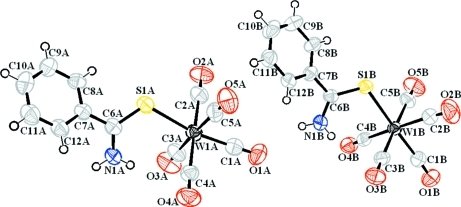
The structure of the title compound with the atomic labelling scheme. Displacement are drawn at the 50% probability level.

**Fig. 2.**
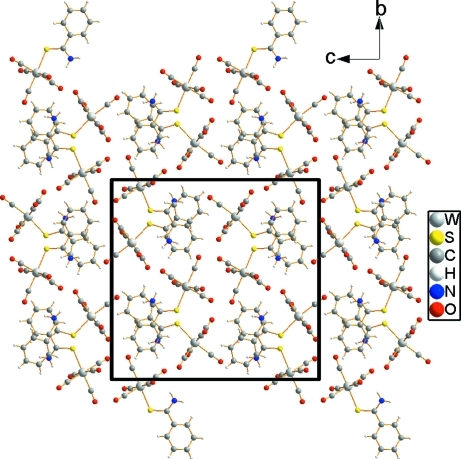
A diagram of the layered crystal packing in (I), viewed down the a axis, showing ondulate layers.

**Fig. 3.**
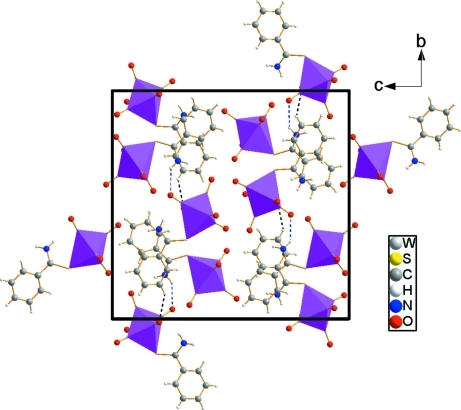
A part of crystal packing showing W(CO)5S octahedral and demeric chains along the b axis. Hydrogen bonds are shown as dashed lines.

## supplementary crystallographic information

### Comment

Thione containing molecules thioamides are important classes of compounds with a wide variety of applications (Gok & Cetinkaya, 2004). The chemical interest of these molecules lies in the fact that they are multi-functional donors with S and N atoms available for coordination, and their biological interest arises from their structural analogy to thiolated nucleosides. A considerable amount of work has been performed on the synthesis and characterization of metal complexes of thione containing molecules as ligands in the past two decades (RAPER, 1994, 1996, 1997). The diverse properties of the thioamide have been attributed to the coordination ability of the heterocyclic RN—C(S)—NR' thioamide group, as a monodentate ligand, to both metallic and non-metallic elements, leading to stable electron donor–acceptor complexes (Raper *et al.*, 1983). As part of our going studies, we report here the synthesis and crystal structure of the title compound, (I). The molecular structure of (I), and the atomic numbering used, is illustrated in Fig. 1. A l l bond distances and angles are within the ranges of accepted values(Saito *et al.*, 2007; Pasynsky *et al.*, 2007; Darensbourg *et al.*, 1999). The tungsten atom displays octahedral geometry with five CO and the benzenecarbothioamide molecule.

The crystal packing in the title structure can be described by undulate layers of W(CO)_5_ and benzenecarbothioamide parallel to (001)plane (Fig. 2).

In the crystal, the components of the structure are linked *via* intermolecular N—H···O and C—H···O hydrogen bonds to form a dimeric chains along the[010](Fig. 3) and additional stabilization within these layers is provided by weak intramolecular C—H···S, N—H···C interactions and Van Der Walls interactions (Table. 1). These interactions link the molecules within the layers and also link the layers together and reinforcing the cohesion of the structure.

### Experimental

A solution of W(CO)6 (137 mg, 1 mmole) and benzenecarbothioamide (102 mg, 1 mmole) in 40 ml of dry THF was irradiated for 2 h with vigorous stirring. The excess of W(CO)6 was moved by filtration and the solvent was evaporated under reduced pressure. The residue was recrystallized from THF/hexane (1:5 ratio). Bright red crystals were washed three times with portions of hexane, and dried under vacuum. Yield:(22%).

### Refinement

All non-H atoms were refined with anisotropic atomic displacement parameters. All H atoms were localized in Fourier maps but introduced in calculated positions and treated as riding on their parent C and N atoms with C—H = 0.93Å and N—H = 0.86Å and *U*_iso_(H) =1.2(carrier atom). The large residual electronic density near The tungsten atoms has no chemical significance.

### Crystal data

**Table:**

[W(C_7_H_7_NS)(CO)_5_]	*F*(000) = 1728
*M**_r_* = 461.10	*D*_x_ = 2.106 Mg m^−^^3^
Monoclinic, *P*2_1_/*n*	Mo *K*α radiation, λ = 0.71073 Å
*a* = 7.311 (1) Å	Cell parameters from 6264 reflections
*b* = 19.567 (2) Å	θ = 0.4–27.5°
*c* = 20.342 (1) Å	µ = 8.10 mm^−^^1^
β = 91.85 (1)°	*T* = 295 K
*V* = 2908.5 (5) Å^3^	Block, red
*Z* = 8	0.05 × 0.05 × 0.04 mm

### Data collection

**Table:**

Nonius KappaCCD diffractometer	5239 reflections with *I* > 2σ(*I*)
Radiation source: Enraf–Nonius FR590	*R*_int_ = 0.039
graphite	θ_max_ = 27.5°, θ_min_ = 2.1°
Detector resolution: 9 pixels mm^-1^	*h* = −9→9
CCD rotation images, thick slices scans	*k* = −25→25
11978 measured reflections	*l* = −26→26
6616 independent reflections	

### Refinement

**Table:**

Refinement on *F*^2^	Secondary atom site location: difference Fourier map
Least-squares matrix: full	Hydrogen site location: inferred from neighbouring sites
*R*[*F*^2^ > 2σ(*F*^2^)] = 0.043	H-atom parameters constrained
*wR*(*F*^2^) = 0.122	*w* = 1/[σ^2^(*F*_o_^2^) + (0.0734*P*)^2^ + 1.0074*P*] where *P* = (*F*_o_^2^ + 2*F*_c_^2^)/3
*S* = 1.09	(Δ/σ)_max_ = 0.002
6616 reflections	Δρ_max_ = 2.05 e Å^−^^3^
362 parameters	Δρ_min_ = −1.81 e Å^−^^3^
0 restraints	Extinction correction: *SHELXL97* (Sheldrick, 1997), Fc^*^=kFc[1+0.001xFc^2^λ^3^/sin(2θ)]^-1/4^
Primary atom site location: structure-invariant direct methods	Extinction coefficient: 0.0038 (2)

### Special details

**Table:**

Geometry. All e.s.d.'s (except the e.s.d. in the dihedral angle between two l.s. planes) are estimated using the full covariance matrix. The cell e.s.d.'s are taken into account individually in the estimation of e.s.d.'s in distances, angles and torsion angles; correlations between e.s.d.'s in cell parameters are only used when they are defined by crystal symmetry. An approximate (isotropic) treatment of cell e.s.d.'s is used for estimating e.s.d.'s involving l.s. planes.
Refinement. Refinement of *F*^2^ against ALL reflections. The weighted *R*-factor *wR* and goodness of fit *S* are based on *F*^2^, conventional *R*-factors *R* are based on *F*, with *F* set to zero for negative *F*^2^. The threshold expression of *F*^2^ > σ(*F*^2^) is used only for calculating *R*-factors(gt) *etc*. and is not relevant to the choice of reflections for refinement. *R*-factors based on *F*^2^ are statistically about twice as large as those based on *F*, and *R*- factors based on ALL data will be even larger.

### Fractional atomic coordinates and isotropic or equivalent isotropic displacement parameters (Å^2^)

**Table:**

	*x*	*y*	*z*	*U*_iso_*/*U*_eq_	
W1B	0.47552 (3)	0.035130 (13)	0.139037 (11)	0.04115 (12)	
W1A	0.50314 (4)	0.184280 (15)	0.597958 (12)	0.04710 (12)	
S1B	0.5287 (2)	0.15399 (8)	0.18654 (7)	0.0438 (3)	
S1A	0.5302 (2)	0.27939 (10)	0.68368 (8)	0.0515 (4)	
C4B	0.2268 (10)	0.0228 (4)	0.1796 (3)	0.0492 (15)	
C11B	0.5994 (11)	0.2913 (4)	0.3958 (4)	0.0592 (18)	
H11B	0.6488	0.2935	0.4384	0.071*	
N1B	0.4325 (8)	0.1066 (3)	0.3024 (3)	0.0503 (13)	
H1B	0.4129	0.1121	0.3435	0.06*	
H2B	0.4178	0.0671	0.2845	0.06*	
O2A	0.2854 (9)	0.2984 (4)	0.5165 (3)	0.0847 (19)	
O3B	0.6891 (8)	−0.0422 (3)	0.2538 (3)	0.0653 (14)	
O4B	0.0839 (7)	0.0142 (3)	0.1981 (3)	0.0650 (14)	
O1A	0.4938 (11)	0.0889 (4)	0.4743 (3)	0.090 (2)	
C1A	0.4951 (12)	0.1235 (5)	0.5205 (4)	0.066 (2)	
O5B	0.2497 (9)	0.1036 (4)	0.0205 (3)	0.089 (2)	
O4A	0.6958 (9)	0.0614 (4)	0.6735 (3)	0.088 (2)	
C7A	0.5009 (8)	0.3088 (4)	0.8138 (3)	0.0458 (14)	
N1A	0.4491 (9)	0.1928 (3)	0.7778 (3)	0.0592 (16)	
H1A	0.4303	0.1828	0.8182	0.071*	
H2A	0.4416	0.1616	0.7481	0.071*	
C11A	0.5770 (12)	0.3401 (5)	0.9253 (4)	0.071 (2)	
H11A	0.6213	0.3284	0.9672	0.085*	
C12A	0.5662 (10)	0.2917 (4)	0.8762 (3)	0.0538 (17)	
H12A	0.6033	0.247	0.885	0.065*	
O1B	0.4266 (10)	−0.1117 (3)	0.0804 (3)	0.0832 (18)	
C6A	0.4889 (8)	0.2556 (4)	0.7615 (3)	0.0469 (15)	
O2B	0.8466 (9)	0.0471 (4)	0.0620 (4)	0.098 (2)	
C4A	0.6293 (10)	0.1075 (4)	0.6486 (4)	0.0543 (17)	
C7B	0.5073 (8)	0.2256 (3)	0.3007 (3)	0.0424 (13)	
C9B	0.4807 (11)	0.3481 (4)	0.2996 (4)	0.0610 (19)	
H9B	0.4475	0.3882	0.2778	0.073*	
C6B	0.4848 (8)	0.1589 (3)	0.2667 (3)	0.0394 (13)	
C8A	0.4464 (9)	0.3749 (4)	0.8014 (3)	0.0497 (15)	
H8A	0.4006	0.3867	0.7598	0.06*	
C8B	0.4610 (9)	0.2857 (4)	0.2682 (3)	0.0495 (15)	
H8B	0.4163	0.2841	0.2249	0.059*	
O5A	0.8865 (8)	0.2385 (4)	0.5503 (3)	0.0842 (19)	
O3A	0.1149 (8)	0.1242 (4)	0.6324 (3)	0.088 (2)	
C10A	0.5207 (13)	0.4069 (5)	0.9116 (4)	0.076 (2)	
H10A	0.5255	0.4397	0.9447	0.091*	
C5A	0.7517 (10)	0.2200 (4)	0.5684 (4)	0.0580 (18)	
C10B	0.5493 (11)	0.3505 (5)	0.3630 (4)	0.067 (2)	
H10B	0.5623	0.3925	0.3841	0.08*	
C3B	0.6128 (9)	−0.0127 (4)	0.2132 (3)	0.0472 (14)	
C2A	0.3655 (11)	0.2584 (4)	0.5450 (4)	0.0596 (18)	
C12B	0.5759 (9)	0.2286 (4)	0.3650 (3)	0.0489 (15)	
H12B	0.606	0.1885	0.3875	0.059*	
C9A	0.4588 (12)	0.4241 (5)	0.8501 (4)	0.066 (2)	
H9A	0.4249	0.4689	0.8407	0.079*	
C1B	0.4418 (11)	−0.0564 (4)	0.0996 (4)	0.0570 (17)	
C2B	0.7157 (11)	0.0451 (4)	0.0899 (4)	0.0576 (18)	
C5B	0.3328 (10)	0.0813 (4)	0.0619 (3)	0.0555 (17)	
C3A	0.2517 (11)	0.1457 (5)	0.6225 (4)	0.063 (2)	

### Atomic displacement parameters (Å^2^)

**Table:**

	*U*^11^	*U*^22^	*U*^33^	*U*^12^	*U*^13^	*U*^23^
W1B	0.04647 (17)	0.03768 (18)	0.03916 (16)	−0.00108 (10)	−0.00078 (10)	−0.00048 (9)
W1A	0.04694 (18)	0.0514 (2)	0.04283 (17)	0.00581 (11)	−0.00016 (11)	0.00570 (11)
S1B	0.0522 (8)	0.0374 (8)	0.0417 (7)	−0.0039 (7)	0.0004 (6)	−0.0012 (6)
S1A	0.0554 (9)	0.0531 (11)	0.0461 (8)	−0.0015 (8)	0.0011 (7)	0.0055 (7)
C4B	0.054 (4)	0.040 (4)	0.053 (4)	−0.003 (3)	0.002 (3)	−0.005 (3)
C11B	0.065 (4)	0.060 (5)	0.052 (4)	−0.009 (4)	0.005 (3)	−0.008 (3)
N1B	0.063 (3)	0.045 (3)	0.043 (3)	−0.004 (3)	0.008 (2)	−0.004 (2)
O2A	0.089 (4)	0.074 (4)	0.089 (4)	0.016 (3)	−0.017 (3)	0.031 (3)
O3B	0.063 (3)	0.066 (4)	0.066 (3)	0.008 (3)	−0.012 (3)	0.016 (3)
O4B	0.054 (3)	0.056 (3)	0.086 (4)	−0.004 (3)	0.016 (3)	0.003 (3)
O1A	0.126 (6)	0.073 (4)	0.070 (4)	0.011 (4)	−0.001 (4)	−0.022 (3)
C1A	0.076 (5)	0.061 (5)	0.060 (5)	0.008 (4)	0.002 (4)	0.007 (4)
O5B	0.097 (5)	0.103 (5)	0.064 (3)	0.003 (4)	−0.022 (3)	0.029 (3)
O4A	0.087 (4)	0.083 (5)	0.095 (4)	0.032 (4)	0.004 (4)	0.032 (4)
C7A	0.039 (3)	0.049 (4)	0.049 (3)	−0.005 (3)	0.002 (3)	0.003 (3)
N1A	0.079 (4)	0.052 (4)	0.048 (3)	−0.008 (3)	0.017 (3)	0.005 (3)
C11A	0.077 (5)	0.082 (7)	0.054 (4)	−0.001 (5)	−0.007 (4)	0.007 (4)
C12A	0.056 (4)	0.058 (5)	0.047 (4)	−0.005 (3)	−0.003 (3)	0.006 (3)
O1B	0.118 (5)	0.055 (4)	0.077 (4)	−0.009 (4)	0.007 (4)	−0.017 (3)
C6A	0.036 (3)	0.055 (4)	0.050 (3)	−0.001 (3)	0.004 (2)	0.005 (3)
O2B	0.069 (4)	0.130 (7)	0.099 (5)	0.004 (4)	0.037 (4)	0.011 (4)
C4A	0.055 (4)	0.050 (4)	0.059 (4)	0.006 (3)	0.004 (3)	0.003 (3)
C7B	0.039 (3)	0.041 (4)	0.047 (3)	−0.001 (3)	0.001 (2)	−0.006 (3)
C9B	0.065 (4)	0.041 (4)	0.077 (5)	0.006 (3)	−0.003 (4)	−0.005 (4)
C6B	0.033 (3)	0.037 (3)	0.048 (3)	−0.001 (2)	0.003 (2)	0.004 (3)
C8A	0.046 (3)	0.047 (4)	0.056 (4)	0.003 (3)	0.000 (3)	0.008 (3)
C8B	0.049 (4)	0.041 (4)	0.057 (4)	0.001 (3)	−0.009 (3)	−0.006 (3)
O5A	0.058 (3)	0.116 (6)	0.080 (4)	−0.009 (4)	0.021 (3)	0.001 (4)
O3A	0.055 (3)	0.112 (6)	0.097 (5)	−0.015 (4)	−0.006 (3)	0.028 (4)
C10A	0.086 (6)	0.080 (7)	0.061 (5)	−0.003 (5)	−0.002 (4)	−0.019 (4)
C5A	0.058 (4)	0.065 (5)	0.051 (4)	0.005 (4)	0.008 (3)	−0.002 (3)
C10B	0.070 (5)	0.054 (5)	0.077 (5)	−0.017 (4)	0.003 (4)	−0.025 (4)
C3B	0.050 (3)	0.039 (3)	0.052 (4)	0.000 (3)	0.002 (3)	0.002 (3)
C2A	0.057 (4)	0.067 (5)	0.054 (4)	−0.003 (4)	−0.003 (3)	0.007 (4)
C12B	0.055 (4)	0.045 (4)	0.047 (3)	−0.006 (3)	0.009 (3)	0.002 (3)
C9A	0.082 (5)	0.058 (5)	0.057 (4)	−0.006 (4)	0.000 (4)	−0.008 (4)
C1B	0.064 (4)	0.054 (5)	0.053 (4)	−0.001 (4)	0.003 (3)	−0.003 (3)
C2B	0.067 (5)	0.051 (4)	0.056 (4)	0.003 (3)	0.008 (3)	0.000 (3)
C5B	0.060 (4)	0.060 (5)	0.046 (4)	0.001 (4)	0.000 (3)	0.008 (3)
C3A	0.059 (4)	0.077 (6)	0.052 (4)	−0.003 (4)	−0.007 (3)	0.014 (4)

### Geometric parameters (Å, °)

**Table:**

W1B—C1B	1.975 (8)	C7A—C12A	1.382 (9)
W1B—C3B	2.016 (7)	C7A—C6A	1.490 (10)
W1B—C4B	2.036 (7)	N1A—C6A	1.308 (9)
W1B—C2B	2.058 (8)	N1A—H1A	0.86
W1B—C5B	2.064 (7)	N1A—H2A	0.86
W1B—S1B	2.5436 (16)	C11A—C12A	1.379 (12)
W1A—C1A	1.974 (9)	C11A—C10A	1.396 (14)
W1A—C4A	2.027 (8)	C11A—H11A	0.93
W1A—C2A	2.052 (8)	C12A—H12A	0.93
W1A—C5A	2.055 (8)	O1B—C1B	1.155 (10)
W1A—C3A	2.063 (8)	O2B—C2B	1.129 (9)
W1A—S1A	2.5537 (19)	C7B—C8B	1.386 (9)
S1B—C6B	1.674 (6)	C7B—C12B	1.387 (9)
S1A—C6A	1.686 (7)	C7B—C6B	1.484 (9)
C4B—O4B	1.134 (8)	C9B—C10B	1.368 (11)
C11B—C10B	1.381 (12)	C9B—C8B	1.383 (10)
C11B—C12B	1.385 (10)	C9B—H9B	0.93
C11B—H11B	0.93	C8A—C9A	1.381 (11)
N1B—C6B	1.318 (8)	C8A—H8A	0.93
N1B—H1B	0.86	C8B—H8B	0.93
N1B—H2B	0.86	O5A—C5A	1.123 (9)
O2A—C2A	1.126 (9)	O3A—C3A	1.110 (9)
O3B—C3B	1.138 (8)	C10A—C9A	1.360 (12)
O1A—C1A	1.157 (10)	C10A—H10A	0.93
O5B—C5B	1.112 (9)	C10B—H10B	0.93
O4A—C4A	1.136 (9)	C12B—H12B	0.93
C7A—C8A	1.375 (10)	C9A—H9A	0.93
			
C1B—W1B—C3B	86.3 (3)	H1A—N1A—H2A	120
C1B—W1B—C4B	87.5 (3)	C12A—C11A—C10A	119.3 (7)
C3B—W1B—C4B	94.1 (3)	C12A—C11A—H11A	120.4
C1B—W1B—C2B	89.2 (3)	C10A—C11A—H11A	120.4
C3B—W1B—C2B	89.8 (3)	C11A—C12A—C7A	120.6 (8)
C4B—W1B—C2B	174.8 (3)	C11A—C12A—H12A	119.7
C1B—W1B—C5B	92.0 (3)	C7A—C12A—H12A	119.7
C3B—W1B—C5B	178.3 (3)	N1A—C6A—C7A	118.9 (6)
C4B—W1B—C5B	85.6 (3)	N1A—C6A—S1A	123.1 (6)
C2B—W1B—C5B	90.4 (3)	C7A—C6A—S1A	118.0 (5)
C1B—W1B—S1B	177.7 (2)	O4A—C4A—W1A	175.1 (7)
C3B—W1B—S1B	94.2 (2)	C8B—C7B—C12B	119.3 (6)
C4B—W1B—S1B	94.67 (19)	C8B—C7B—C6B	120.2 (6)
C2B—W1B—S1B	88.6 (2)	C12B—C7B—C6B	120.5 (6)
C5B—W1B—S1B	87.5 (2)	C10B—C9B—C8B	119.7 (8)
C1A—W1A—C4A	87.7 (3)	C10B—C9B—H9B	120.2
C1A—W1A—C2A	90.3 (3)	C8B—C9B—H9B	120.2
C4A—W1A—C2A	177.0 (3)	N1B—C6B—C7B	117.1 (6)
C1A—W1A—C5A	88.6 (3)	N1B—C6B—S1B	124.1 (5)
C4A—W1A—C5A	90.5 (3)	C7B—C6B—S1B	118.9 (5)
C2A—W1A—C5A	91.7 (3)	C7A—C8A—C9A	120.8 (7)
C1A—W1A—C3A	88.2 (4)	C7A—C8A—H8A	119.6
C4A—W1A—C3A	90.0 (3)	C9A—C8A—H8A	119.6
C2A—W1A—C3A	87.7 (3)	C9B—C8B—C7B	120.6 (7)
C5A—W1A—C3A	176.7 (3)	C9B—C8B—H8B	119.7
C1A—W1A—S1A	169.8 (3)	C7B—C8B—H8B	119.7
C4A—W1A—S1A	99.7 (2)	C9A—C10A—C11A	120.2 (8)
C2A—W1A—S1A	82.5 (2)	C9A—C10A—H10A	119.9
C5A—W1A—S1A	84.4 (2)	C11A—C10A—H10A	119.9
C3A—W1A—S1A	98.7 (3)	O5A—C5A—W1A	177.7 (7)
C6B—S1B—W1B	113.0 (2)	C9B—C10B—C11B	120.6 (7)
C6A—S1A—W1A	115.3 (3)	C9B—C10B—H10B	119.7
O4B—C4B—W1B	175.2 (6)	C11B—C10B—H10B	119.7
C10B—C11B—C12B	119.9 (7)	O3B—C3B—W1B	177.2 (6)
C10B—C11B—H11B	120.1	O2A—C2A—W1A	178.0 (7)
C12B—C11B—H11B	120.1	C11B—C12B—C7B	119.9 (7)
C6B—N1B—H1B	120	C11B—C12B—H12B	120
C6B—N1B—H2B	120	C7B—C12B—H12B	120
H1B—N1B—H2B	120	C10A—C9A—C8A	120.0 (8)
O1A—C1A—W1A	178.2 (9)	C10A—C9A—H9A	120
C8A—C7A—C12A	119.1 (7)	C8A—C9A—H9A	120
C8A—C7A—C6A	121.1 (6)	O1B—C1B—W1B	175.5 (7)
C12A—C7A—C6A	119.8 (7)	O2B—C2B—W1B	176.3 (7)
C6A—N1A—H1A	120	O5B—C5B—W1B	176.5 (7)
C6A—N1A—H2A	120	O3A—C3A—W1A	176.3 (7)

### Hydrogen-bond geometry (Å, °)

**Table:**

*D*—H···*A*	*D*—H	H···*A*	*D*···*A*	*D*—H···*A*
N1A—H2A···O3B^i^	0.86	2.52	3.174 (8)	133
C9B—H9B···O4B^ii^	0.93	2.53	3.285 (10)	139
C8A—H8A···S1A	0.93	2.79	3.114 (7)	101
C8B—H8B···S1B	0.93	2.79	3.114 (6)	101
N1B—H2B···C3B	0.86	2.59	3.263 (9)	136
N1A—H2A···C4A	0.86	2.69	3.412 (10)	142

Symmetry codes: (i) −*x*+1, −*y*, −*z*+1; (ii) −*x*+1/2, *y*+1/2, −*z*+1/2.
